# Immediate Breast Reconstruction with a Deep Inferior Epigastric Perforator Flap in the Lithotomy Position

**DOI:** 10.1097/GOX.0000000000002552

**Published:** 2019-12-26

**Authors:** Shihoko Tamura, Toshihiko Satake, Mayu Muto, Mai Shibuya, Kazutaka Narui, Shinji Kobayashi, Takashi Ishikawa, Jiro Maegawa

**Affiliations:** From the * Department of Plastic and Reconstructive Surgery, Yokohama City University Medical Center, Yokohama, Kanagawa, Japan; † Department of Breast and Thyroid Surgery, Yokohama City University Medical Center, Yokohama, Kanagawa, Japan; ‡ Department of Plastic and Reconstructive Surgery, Kanagawa Children’s Medical Center, Yokohama, Kanagawa, Japan; § Department of Breast Oncology and Surgery, Tokyo Medical University Hospital, Shinjuku, Tokyo, Japan; ¶ Department of Plastic and Reconstructive Surgery, Yokohama City University Hospital, Yokohama, Kanagawa, Japan.

## Abstract

Mastectomy and flap harvesting can be accomplished simultaneously in immediate deep inferior epigastric perforator (DIEP) flap breast reconstruction. However, this is not always possible, particularly in a teaching institution, where supervisors, trainees, and assistants must participate in the surgery, because there is not enough working space for breast and plastic surgeons to perform surgery together. We attempted to overcome this problem by placing the patient in the lithotomy position and have reported the outcomes. We evaluated patients who underwent unilateral immediate DIEP flap breast reconstruction in the supine or lithotomy position between October 2014 and July 2016. The surgeries were performed by the same inexperienced plastic surgeon in our hospital. In the lithotomy position, 1 plastic surgeon stands between the patient’s legs and 1 stands beside the abdomen, and they perform DIEP flap harvesting simultaneously with mastectomy performed by 3 breast surgeons. After mastectomy, breast reconstruction is performed by 4 plastic surgeons. The supine position was used in the first 8 patients, and the lithotomy position was used in the following 8 patients. The mean operative time was 11 hours 21 minutes in the supine group and 8 hours 52 minutes in the lithotomy group, with a significant difference (*P* = 0.027). Breast reconstruction with a DIEP flap in the lithotomy position is useful for teaching institutions because it provides sufficient working space and allows simultaneous procedures without prolonging operative time. However, issues such as pressure sores, nerve palsy, and difficulty in patient placement still exist.

## Abstract 2

Supplemental Digital Content is available in the text.

## INTRODUCTION

Breast reconstruction with a deep inferior epigastric perforator (DIEP) flap is typically performed in the supine position. Reportedly, concurrent mastectomy and abdominal flap elevation can be accomplished in this position.^1^ However, in teaching institutions, we found it difficult to perform the surgery with a 2-team approach. When 3 breast surgeons (a supervisor, a trainee, and an assistant) perform mastectomy in the supine position, there is not enough working space for the plastic surgeon and his assistant to harvest the DIEP flap. Therefore, plastic surgeons initiate harvesting only after the breast surgeons complete mastectomy. To create more working space, we used the lithotomy position and have reported the outcomes here.

## PATIENTS AND METHODS

We compared patients who underwent unilateral immediate DIEP flap breast reconstruction in the supine and lithotomy positions between October 2014 and July 2016 at Yokohama City University Medical Center by the same surgeon, who was a plastic surgeon who performed <5 DIEP flap breast reconstructions. All patients were consecutive, and the lithotomy group followed the supine group.

In either position, when nipple-sparing mastectomy (NSM) is performed, the shoulder joint is moved outward, the elbow joint is bent 90-degree, and the forearm is rotated inward to approach the recipient thoracodorsal vessels from the lateral incision. When skin-sparing mastectomy (SSM) is performed, the arms are fixed straight beside the body to approach the internal mammary vessels thorough a round periareolar and transverse skin incision.

**Fig. 1.**
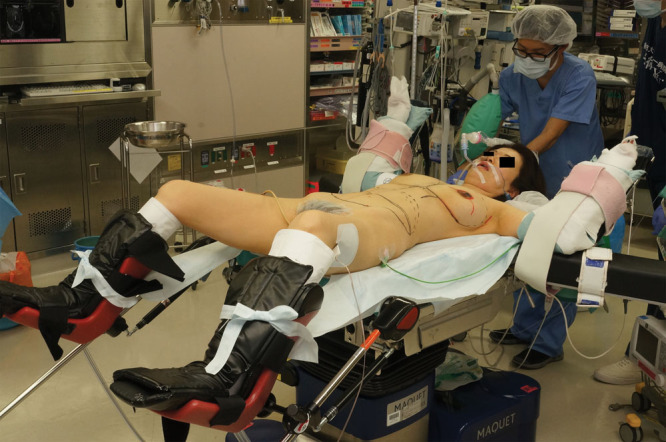
Left mastectomy and immediate one-stage breast reconstruction using a DIEP flap in the lithotomy position without draping. Both upper extremities are positioned with the shoulders in 90-degree abduction, elbows in 90-degree flexion, and forearms in 45-degree medial rotation. The buttock is placed at the lowest end of the operating table. Footstools are used to elevate both legs in the lithotomy position.

**Fig. 2.**
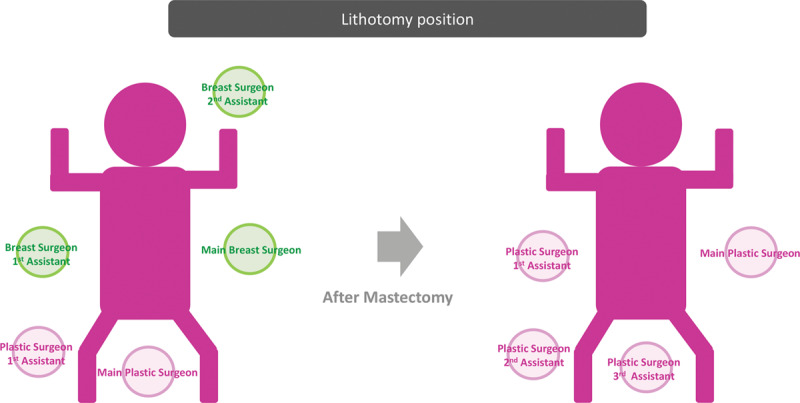
Intraoperative patient position (lithotomy) and surgeon layout for left breast cancer. In the lithotomy position, the main breast surgeon and the first and second assistants stand on the affected side, unaffected side (in front of the main surgeon), and cranial side, respectively, whereas the main plastic surgeon stands between both legs and the first assistant stands beside the right thigh during mastectomy. After mastectomy and flap harvesting, the main and first assistant plastic surgeons move to the cranial side for both microsurgery and flap insetting, and two assistant plastic surgeons stand around the lower abdomen to close the donor site.

In the lithotomy position, the buttock is placed at the lowest end of the operating table. Footstools are used to elevate the legs (**Fig. 1**). Working space is created between the legs for the main plastic surgeon. Three breast surgeons are present in the front (main breast surgeon on the affected side, the supervisor on the cranial side, and an assistant on the unaffected side), the main plastic surgeon stands between the legs, and an assistant stands beside the thigh on the unaffected side (**see figure, Supplemental Digital Content 1**, which shows the intraoperative surgeon layout, http://links.lww.com/PRSGO/B257). After mastectomy, breast reconstruction is performed by 4 plastic surgeons. Two plastic surgeons perform surgery in the breast region (microsurgical anastomosis and flap insetting), and the other 2 plastic surgeons perform surgery in the abdominal region (closing the donor site wound) (**Fig. 2**).

**Fig. 3.**
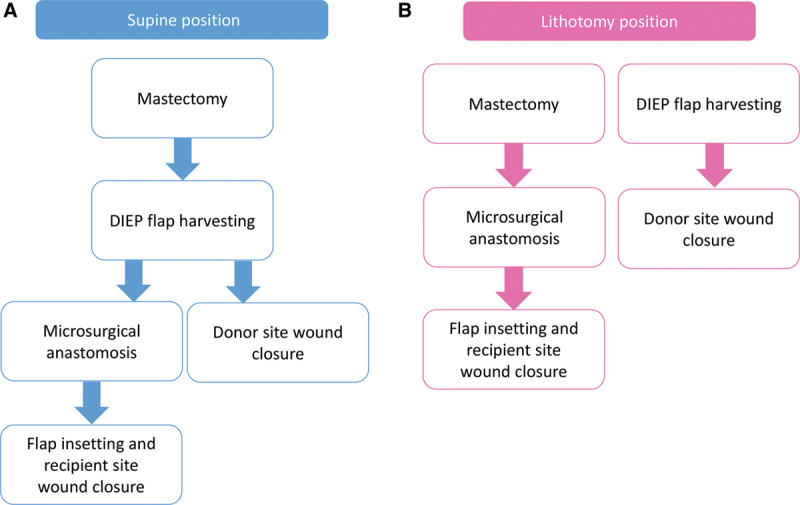
A, In the supine position, plastic surgeons can start DIEP flap harvesting only after mastectomy by breast surgeons. After flap harvesting, 2-team approaches can be used for microsurgical anastomosis, flap insetting, and donor site wound closure. B, In the lithotomy position, breast and plastic surgeons can perform mastectomy and flap harvesting simultaneously from the start of the surgery.

In the supine position, as there is limited space, the surgery starts with only 3 breast surgeons (**see figure, Supplemental Digital Content 2**, which shows the intraoperative surgeon layout in the supine position, http://links.lww.com/PRSGO/B258). In this position, 2-team approaches can be used for microsurgical anastomosis, flap insetting, and donor site wound closure after mastectomy and flap harvesting 1 at a time (**Fig. 3A**). However, in the lithotomy position, because mastectomy and flap harvesting can be simultaneously performed, the operative time can be saved (**Fig. 3B**).

We evaluated patients’ age, height, and weight; mastectomy type; harvested flap and used flap weights; length of stay; and operative time. Student *t*-test was used to analyze continuous variables. *P* ≤ 0.05 was considered statistically significant.

## RESULTS

The supine position was used in the first 8 patients, and the lithotomy position was used in the following 8 patients (Table 1). The mean patient age was 46.5 years in the supine group and 48 years in the lithotomy group. The mean patient height and weight were 158.2 cm and 58.5 kg, respectively, in the supine group and 164.6 cm and 61 kg, respectively, in the lithotomy group. With regard to mastectomy, 3 patients underwent NSM and 5 underwent SSM in the supine group, whereas 5 underwent NSM and 3 underwent SSM in the lithotomy group. The mean harvested flap and used flap weights were 881 g and 478 g, respectively, in the supine group and 719 g and 388 g, respectively, in the lithotomy group. There were no significant differences between the groups. However, the mean operative time was 11 hours 21 minutes in the supine group and 8 hours 52 minutes in the lithotomy group, with a significant difference (*P* = 0.027).

**Table 1.** Patient Demographics in the Supine and Lithotomy Groups

Parameter	Supine group (n = 8)	Lithotomy group (n = 8)	*P*
Mean age	46.5 (38–57)	48 (41–53)	0.89
Mastectomy type	NSM: 3 cases, SSM: 5 cases	NSM: 5 cases, SSM: 3 cases	
Mean height (cm)	158.2 (152–170)	164.6 (152.5–169)	0.52
Mean body weight (kg)	58.5 (49.5–71)	61 (49.5–73)	0.87
Mean flap weight, harvested (g)	881 (578–1,432)	719 (610–1,480)	0.86
Mean flap weight, used (g)	478 (340–628)	388 (254–660)	0.38
Mean length of stay	10 (9–12)	10 (9–13)	0.35
Mean operative time	11 h 21 min	8 h 52 min	0.027

No complication at the reconstructed breast or abdominal donor site was noted in all 16 patients. However, there was a complication related to intraoperative positioning. One patient from the lithotomy group had stage 1 pressure sores at the outer bottoms of both feet. This patient’s operative time was 8 hours 13 minutes. The pressure sore improved within 3 days by conservative therapy.

## DISCUSSION

Breast reconstruction with a DIEP flap in the lithotomy position is useful to ensure sufficient working space and for a 2-team approach. In our study, the operative time was shortened in the lithotomy position than in the supine position; we considered that this was caused by the operator’s learning curve; simultaneous mastectomy and flap harvesting; the addition of co-surgeons, which has been reported to reduce operative time; mean length of stay; and postoperative complications.^2^ We could not evaluate the true length of stay because of the Japanese system called “clinical pass” that standardizes the hospital treatment according to the disease. We expect that the DIEP flap in the lithotomy position will be useful in case of excessively short patients or large operators to gain the working space.

The disadvantages include inability to assess the mounted flap with the patient sitting up straight at 90-degree and complications related to the lithotomy position. Complications include pressure sores, lower limb nerve paralysis, and compartment syndrome. The risk factors for nerve paralysis are operative time >4 hours, patient BMI <20 kg/m^2^, and perioperative smoking history.^3^ Compartment syndrome has mainly been reported in cases with operative time >3 hours.^4,5^ To prevent pressure sores, the lithotomy position should be set by experienced surgeons and decompression should be performed every 2 hours.

To reconstruct a well-shaped breast, we now seek to change the patient’s position to supine when the DIEP flap elevation is complete. The surgery can be resumed within only 10 minutes after changing the patient position, disinfecting the operative field, and redraping the patient with sterilized sheets. This position change enables the assessment of the mounted flap with the patient sitting up straight at 90-degree.

In this study, the number of surgeons performing the immediate DIEP flap breast reconstruction was large (3 breast surgeons and 4 plastic surgeons), which is an uncommon setting. Hence, this procedure can be reproduced in situations where additional surgeons are able to take part in the surgery at teaching institutions, the surgery is to be performed on excessively short patients, or more number of surgeons are available.

## SUMMARY

Breast reconstruction with a DIEP flap in the lithotomy position is useful for teaching institutions with additional operators. It provides sufficient working space and allows simultaneous procedures to be performed.

## ACKNOWLEDGMENTS

The authors would like to thank Dr. Ko Okumura (Department of Plastic and Reconstructive Surgery, Shinko Hospital, Hyogo, Kobe, Japan) for giving us the idea of intraoperative patient position in immediate DIEP flap breast reconstruction. This study respects statement of institutional review board approval and/or statement of conforming to the Declaration of Helsinki.

## Supplementary Material
