# Supplementary figures and images for: Immediate Breast Reconstruction with a Deep Inferior Epigastric Perforator Flap in the Lithotomy Position
Source: Plast Reconstr Surg Glob Open. 2019 Dec 26;7(12):e2552. doi: 10.1097/GOX.0000000000002552 (PMC7288896; doi:10.1097/GOX.0000000000002552)

Supine position

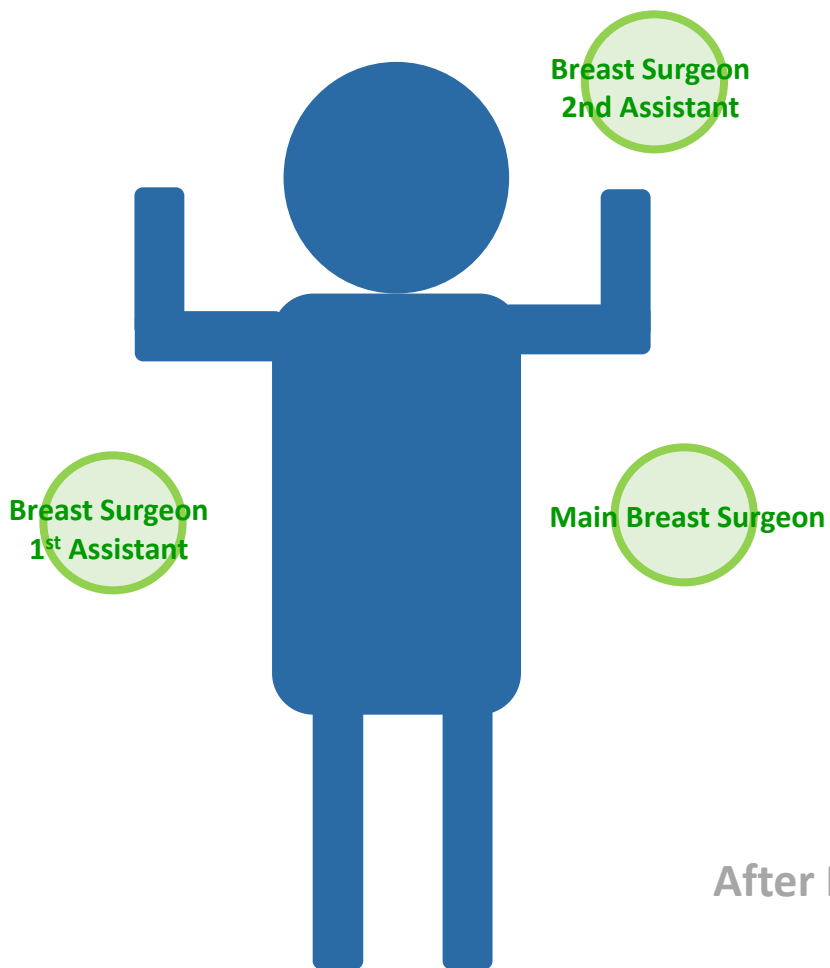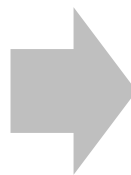

After Mastectomy

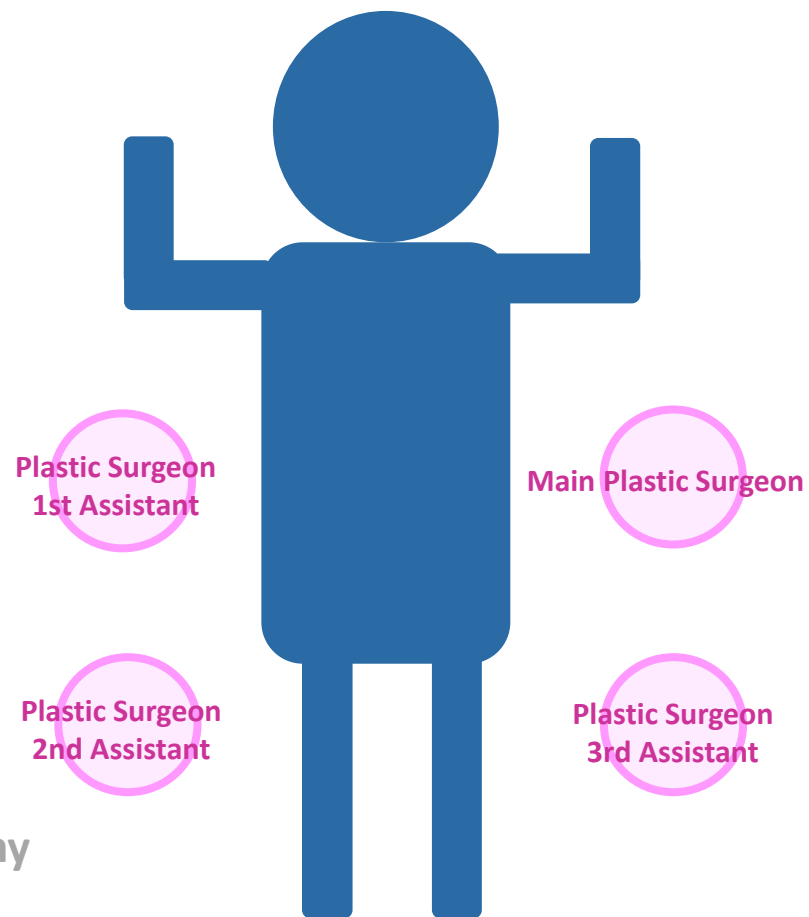

Supplement: Supplementary file 1 [file gox-7-e2552-s001.pdf]
