# Supplementary material for: Immediate Breast Reconstruction with a Deep Inferior Epigastric Perforator Flap in the Lithotomy Position
Source: Plast Reconstr Surg Glob Open. 2019 Dec 26;7(12):e2552. doi: 10.1097/GOX.0000000000002552 (PMC7288896; doi:10.1097/GOX.0000000000002552)
Supplement: Supplementary file 2 [file gox-7-e2552-s002.pdf]

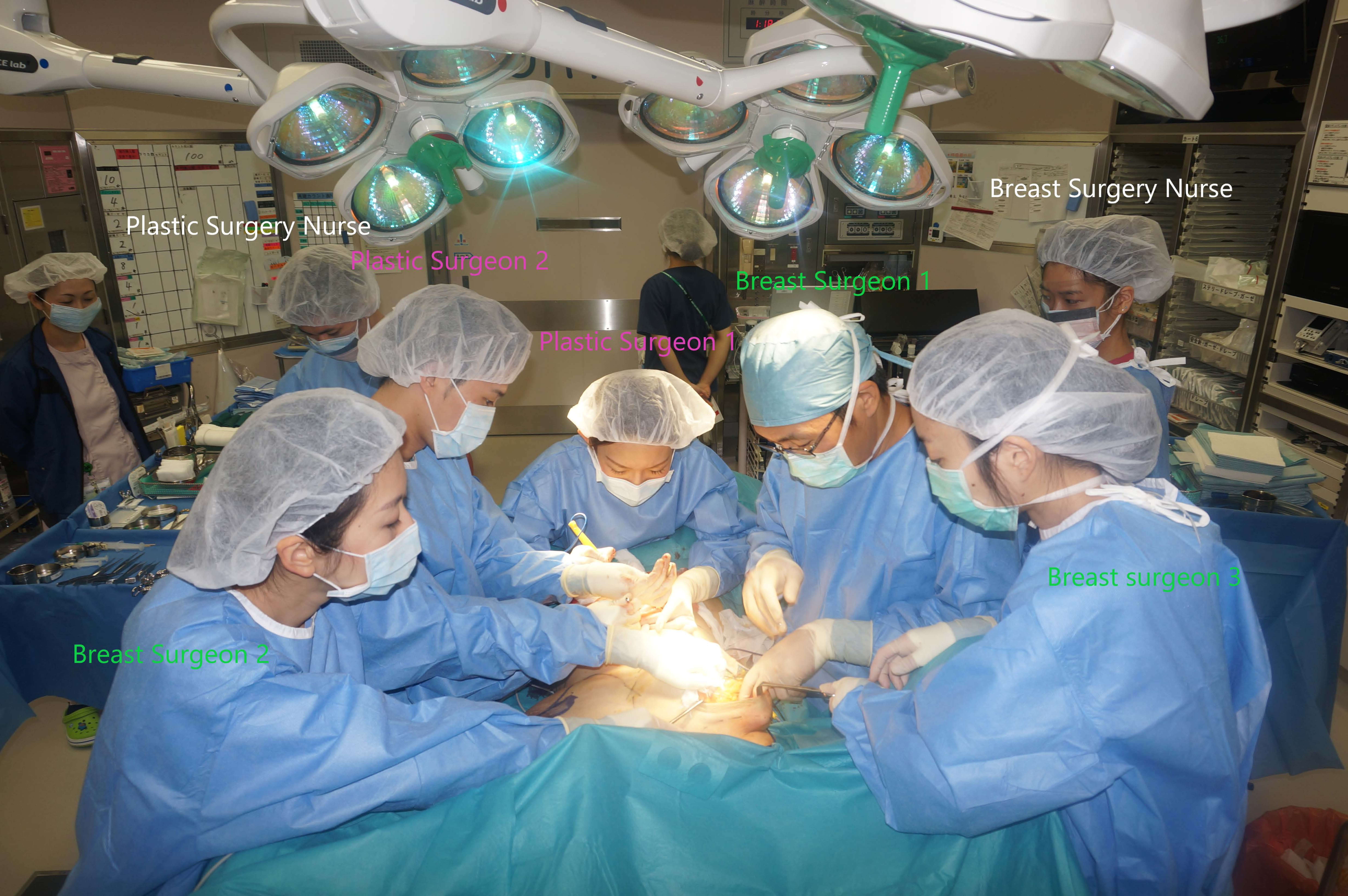
Breast Surgery Nurse
Plastic Surgery Nurse
Plastic Surgeon 2
Breast Surgeon 1
Plastic Surgeon 1
Breast surgeon 3
Breast Surgeon 2
